# Perception of yips among professional Japanese golfers: perspectives from a network modelled approach

**DOI:** 10.1038/s41598-021-99128-9

**Published:** 2021-10-11

**Authors:** Gajanan S. Revankar, Yuta Kajiyama, Yasufumi Gon, Issei Ogasawara, Noriaki Hattori, Tomohito Nakano, Sadahito Kawamura, Yoshikazu Ugawa, Ken Nakata, Hideki Mochizuki

**Affiliations:** 1 Department of Neurology, Graduate School of Medicine, Osaka University, Suita, Osaka 5650871 Japan; 2 Department of Health and Sport Sciences, Graduate School of Medicine, Osaka University, Suita, Osaka 5650871 Japan; 3 Department of Orthopedic Surgery, Graduate School of Medicine, Osaka University, Suita, Osaka 5650871 Japan; 4 Institute for Transdisciplinary Graduate Degree Programs, Osaka University, Suita, Osaka 5650871 Japan; 5 Department of Rehabilitation, Faculty of Medicine, Academic Assembly, University of Toyama, Toyama, 9300194 Japan; 6 Department of Human Neurophysiology, School of Medicine, Fukushima Medical University, Fukushima, 9601295 Japan

**Keywords:** Health care, Neurology, Health occupations

## Abstract

‘Yips’ in golf is a complex spectrum of anxiety and movement-disorder that affects competitive sporting performance. With unclear etiology and high prevalence documented in western literature, the perception and management of this psycho-neuromuscular problem among Japanese elite golfers is unknown. The objective of this study was to explore factors associated with yips, investigate the performance deficits and the strategies implemented to prevent yips. We surveyed approx. 1300 professional golfers on their golfing habits, anxiety and musculoskeletal problems, kinematic deficits, changes in training and their outcomes. Statistical procedures included multiple logistic regression and network analysis. 35% of the respondents had experienced yips in their career, their odds increasing proportionally to their golfing experience. Regardless of musculoskeletal symptoms, about 57% of all yips-golfers attributed their symptoms to psychological causes. Network analysis highlighted characteristic movement patterns, i.e. slowing, forceful or freezing of movement for putting, approach and teeing shots respectively. Golfers’ self-administered strategies to relieve yips were mostly inconsequential. Within the limits of our self-reported survey, most golfers perceived yips as a psychological phenomenon despite evidence pointing to a movement-disorder. While self-administered interventions were satisfactory at best, it may be imperative to sensitize golfers from a movement-disorder standpoint for early management of the problem.

## Introduction

Yips in professional golfers presents an acute involuntary loss of motor skill during high-pressure sporting environments^1,2^. The impact of such performance deficit often leads to athletes abandoning the game^1^. Not limited only to golf, yips affects other sports as well, some of which include cricket, baseball, archery and snooker^3^.

Presently, the exact origin of yips is still undefined but is known to lie on a continuum between competition anxiety and task-specific movement disorder^4–6^. The anxiety component generally triggers as ‘choking’ wherein athletes cannot execute an action because of their perception of attentional resources as being insufficient^7^. This relates to how sports anxiety is defined, i.e. an unpleasant state of stress resulting in under-performance^8^. As a result, yips is considered a neuropsychological phenomenon because of its congruity with high-pressure situations and its near-complete absence of symptoms in day-to-day activities. On the other hand, ‘yips’ is also suggested to be a movement disorder characterized by abnormal, involuntary, twitching, jerks, spasms or freezing of planned motor movement in repeatedly learnt skilled activity^9,10^. In literature, this motor activity has been revealed as an abnormal co-contraction of specific upper limb musculature, and therefore has been characterized as a form of task-specific focal dystonia akin to musician’s dystonia or writer’s cramp^6,11^. Among yips golfers, these co-contractions are known to impact the musculoskeletal system thereby affecting the kinematic outcomes of gameplay^12–14^.

In practice however, golfers usually fall somewhere in between these two pure subtypes (anxiety or dystonia) of the yips spectrum^3–6^. With several overlapping features, yips is a diagnostic gray zone with one type influencing the other^11^. This has motivated researchers to theorize alternative explanations that suggest yips to be a contextual movement disturbance^4^, a mixture of focal dystonia and anxiety^3,4^, or stereotyped behavior that interferes with automatized movements^15^. Additionally, these golfers are also known to be affected by personality traits that indicate a degree of social anxiety and perfectionism making them susceptible to choking^5,16^.

Considering the above aspects, the overall prevalence of yips is therefore considerably higher than that of task-specific focal dystonia (e.g. musician’s dystonia affects about 1% of all professional musicians^17^). Prior studies have reported anywhere between 20 and 48% of elite professional golfers to be affected by the yips^18–20^. Despite such high prevalence, most golfers self-manage their performance issues by changing grip style, or putter length to avoid aggravating their symptoms^18^. However, details of such self-care interventions are unknown. Furthermore, it is currently unclear to what extent golfers change their training habits, receive professional medical and/or psychological support to relieve yips symptoms.

Given the diverse etiology, prevalence and factors involved in the precipitation and maintenance of yips, we aimed to address these issues in an expansive manner with the use of self-reported surveys. Self-reporting is considered to be a valuable method in obtaining subjects’ perspectives and evaluating participant opinions in terms of their physical condition^21^. While several researchers have performed well-designed experimental work to define the pathophysiological mechanisms of yips^4,6,22,23^, studies have also employed questionnaires or semi-structured interviews to characterize the spectrum of yips^11,18–20^. Notably, Smith et al. performed a focused investigation among yips-only golfers to subjectively evaluate their ‘perception’ regarding yips^24^. The rationale behind this exercise was to observe the influence of self-perception on their symptoms, their golfing habits and their compliance towards getting professional support (psychological, neurological or orthopedic) regarding the performance deficit. This, and several others studies focusing on western populations^2,19,23^, provided a promising approach to divulge yips symptoms via participant derived responses to facilitate management of such golfers.

Equipped with the above information, we analyzed a large-scale, cross-sectional survey among golfers in Japan, the groundwork formulated previously on amateur and highly skilled professional golfers^25^, to ascertain their demographic characteristics, golfing habits, their experience with yips and the kinematic issues associated with it. The objective of this self-reported survey was to explore factors associated with yips and assess how elite golfers in Japan perceive this psycho-neuromuscular phenomenon. We also investigated what strategies golfers implemented to relieve their symptoms and evaluate the outcomes of such measures. Our aim of this study was therefore to present a systematic characterization of yips in Japanese professional golfers, literature of which is currently sparse. Beyond the qualitative analysis, we also exploited network methodologies to analyze the survey dataset. Prior studies have highlighted the potential advantages of incorporating logistic regression through network analysis in neuropsychiatric disease datasets to demonstrate the relationships between different symptoms^26–28^. We applied this concept to our data since network structures provide an efficient modality to examine interactions between large number of variables^29,30^.

## Methods

### Design and population

We surveyed participants from the Professional Golfers' Association (PGA), Japan, focusing specifically on elite golfers with a professional teaching license (median interquartile range of handicap score of 0 (0, 0)). The study was performed between 2016 and 2018 wherein golfing information was collected from respondents aged 18 and above. Participants were recruited via in-person seminar sessions and training workshops that were held in PGA. The paper-based survey questionnaires were handed out to 1356 participants. Prior to its distribution, a movement-disorder specialist primed the golfers regarding the features, characteristics and the spectrum of yips. The research was performed under all the relevant guidelines and regulations, and under the Declaration of Helsinki. The Osaka University institutional review board (IRB) for clinical research approved this study. Informed consent was obtained from all the golfers who participated in the survey.

### Survey measures and outcomes

Demographic and golfing details in the questionnaire included age, gender, years of golfing experience, handedness, practice hours per month, number of golfing rounds per year and number of private practice rounds. Questions related to musculoskeletal problems were provided with multiple responses with a severity scale that encompassed movement problems during competitions (low), during practice and competitive golf (moderate), and those that affected activities of daily living (severe). Golfers described any fine motor activities that affected their daily life in a free-range manner (e.g. difficulties in balancing glass full of water, stacking items, any uncoordinated hand-eye movements etc.). In order to screen for anxiety or nervousness trait, we asked participants whether their personality was of anxious type in public (situations) and whether they attributed their yips symptoms to competition anxiety. Based on a single question that asked whether the golfers suffered from yips or not, additional questions followed. Such of those who responded positively for presence of yips, details regarding their speculated cause of yips (a movement disorder, or a psychological disorder or something else), their problem type related to club use (wood, iron, wedge or putter) and problems in shot type (tee, fairway, driver, rough, bunker, approach and putt) was obtained. To define the movement problems of the golfers, we documented them under a pre-defined list of five most common types, since feature definitions from free-response questions have been extremely diverse in yips golfers^24^. These included forceful shots, sluggish/slowing of shots, tremors, jerks and freezing. Last, participants were requested to provide details of their strategies to relieve the symptoms of yips that listed changes in golfing techniques (e.g. grip changes, use of gloves, changing length/size of the clubs, handedness, etc. i.e. free-responses), increasing or decreasing the frequency of training (practice hours), and the outcome of these changes (improved, worsened or no change). Based on this formulation, we reported the differences between normal and yips golfers and an exploratory analysis of the influence of such variables among yips only group.

### Statistical analysis

Descriptive statistics were reported as mean and standard deviations (SD) for relevant demographic variables. Missing values for demographic data were imputed using nearest neighbor method (knnimpute function on Matlab). Missing values counts were, for age n = 1, for experience n = 2, for practice hours n = 30, and for rounds/year n = 38. To test the differences between the normal and yips group, non-parametric Mann–Whitney test was performed with statistical significance set to *p* < 0.05. To ascertain which of the demographic factors were associated with yips, we opted for a multivariate logistic regression analysis specifying a binomial distribution representing the 2 groups (with and without yips). The proportional odds ratio and confidence intervals for the above variables were reported for *p* < 0.05 (Wald’s statistic). To evaluate anxiety and severity of musculoskeletal problems between the groups, a Chi-square test with alpha = 0.05 was performed along with graphical representation of frequency estimates.

A subgroup analysis was performed for yips only group. Frequency estimates were calculated for club-type, shot-type, movement disorder-type and training-type categories. Considering the large number of variables, we used network analysis to gather a holistic picture of yips golfers’ characteristics. In brief, a network comprises several ‘nodes’ of interest and the link between these nodes are defined by its ‘edges’^31^. With reference to our dataset, each dependent variable served as a ‘node’, whereas ‘edges’ represented the relationship between the variables after conditioning other variables. Considering the categorical binary nature of our data, we applied a recently developed Ising model of logistic regression to study the network structure. Ising models have shown to be a promising approach to study large psychological datasets that are predominantly populated with ‘yes’/‘no’ type data^32^. Network analysis was performed on shot-type, movement problem-type and training-type using IsingFit function on JASP statistical software. Club-type estimates were excluded because of high collinearity with shot-type data. Edge parameters rules included: non-zero regression coefficients between nodes (‘AND’ rule) and gamma hyperparameter value at 0.25. To ascertain the accuracy of edge-weights^33^, we bootstrapped the edges of the estimated network and their centrality on 1000 random networks. Between-node edge weight confidence intervals which did not overlap with other non-zero nodes were considered significant. Nodes in the network were positioned via the Fruchterman–Reingold algorithm wherein the structure was defined based on the nodal connection strength^34^. When reporting the effects between different outcomes, network structures were constrained for clear visualization and comparability. Subsequent network properties were then analyzed with respect to degree centrality, betweenness and expected influence of nodal properties^35^. Degree centrality defined the importance of the variable in the network, and was illustrated by the number of connections of that node to all other nodes in the graph^36^. Betweenness centrality provided a quantification of the node serving as a bridge between two other connected nodes along its shortest path. Finally, in a network with positive and negative edges, the expected influence metric factored in the negative associations among the nodes for interpretations on the variable importance^35^.

All preprocessing of data was performed on Matlab v2018b (MathWorks, USA). Statistical analysis and graphing were done on JASP software (v0.14) that uses relevant R packages (qgraph) for network analysis. For statistical tests, *p* values lower than 0.05 were considered significant. Data analyzed in this manuscript will be made available from the corresponding author upon reasonable request.

## Results

Demographics characteristics of the respondents are described in Table 1. We obtained 1271 (out of 1356) participant responses from the questionnaire, achieving a 94% response rate for the survey.

**Table 1:** Demographic characteristics of professional golfers.

	1. Total respondents (n = 1271)		2. No reported symptoms of yips (n = 821)		3. Have experienced yips (n = 450)		*p* value	Effect size (rank-based correlation)
	%	n	%	n	%	n	2 versus 3	
Age	48.2 ± 9.8	1270	47.7 ± 10.0	820	49.0 ± 9.5	450	**0.019**	− 0.08
﻿Age group								
*18–29*	1%	15	1%	12	< 1%	3		
*30–39*	14%	176	15%	123	12%	53		
*40–49*	37%	475	38%	314	36%	161		
*50–64*	41%	514	38%	312	45%	202		
*> 65*	7%	91	7%	60	7%	31		
Handedness								
*Right*	89%	1133	90%	737	88%	396		
*Left*	4%	50	4%	32	4%	18		
*Not responded*	7%	90	6%	52	8%	38		
Experience	29.8 ± 9.9		29.1 ± 10.0		31.2 ± 9.7		**< 0.001**	− 0.13
Practice hours/month	2.8 ± 2.9		2.8 ± 3.0		2.8 ± 2.6		0.29	–
Rounds per year	47.8 ± 38.4		48.5 ± 39.0		46.4 ± 37.2		0.29	–

Reported *p* values are derived from Mann–Whitney non-parametric test. Significant *p* values are shown in bold.

35.4% of the respondents acknowledged to have had yips during their career (N = 450). Most yips golfers (approx. 45%) were in the 50 to 64-year age category. Age and experience between yips and without yips participants, though statistically significant, were roughly similar (effect sizes approx. 0.1, rank-based correlation).

**Figure 1:**
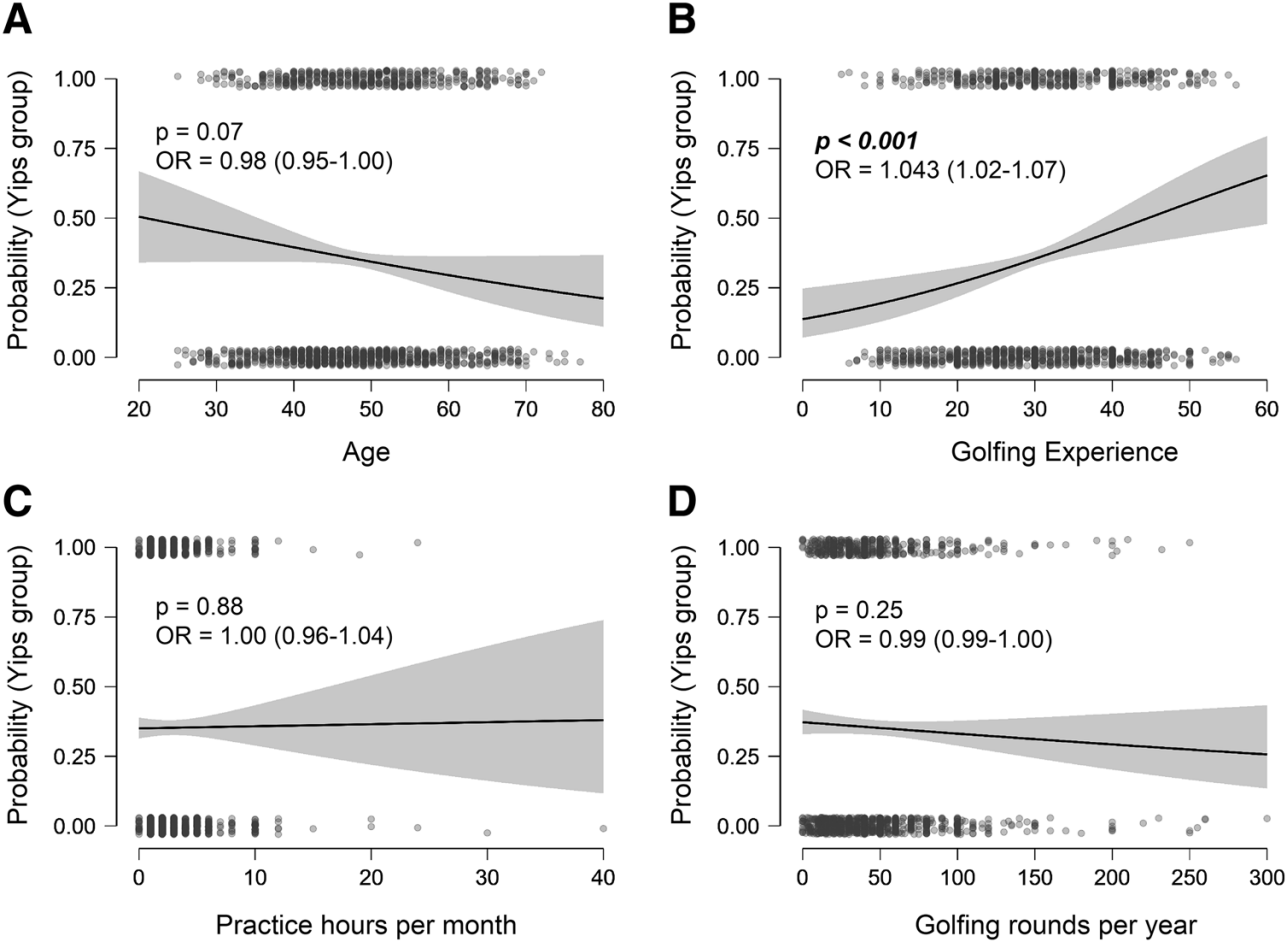
Multiple logistic regression of demographic predictors for yips. Conditional estimates plot for (**A**) age, (**B**) golfing experience, (**C**) practice hours/month and (**D**) rounds/year. OR = Odds ratio with confidence intervals for odds ratio. Yips groups coded as 1. Golfers with yips: N = 450, golfers without yips: N = 821.

Multivariate logistic regression analysis of the binomial variable ‘Group’, given the reference level of all other factors, showed golfing experience to be a significant predictor in the occurrence of yips (OR, CI = 1.043, 1.02–1.07, *p* < 0.001), Fig. 1.

**Figure 2:**
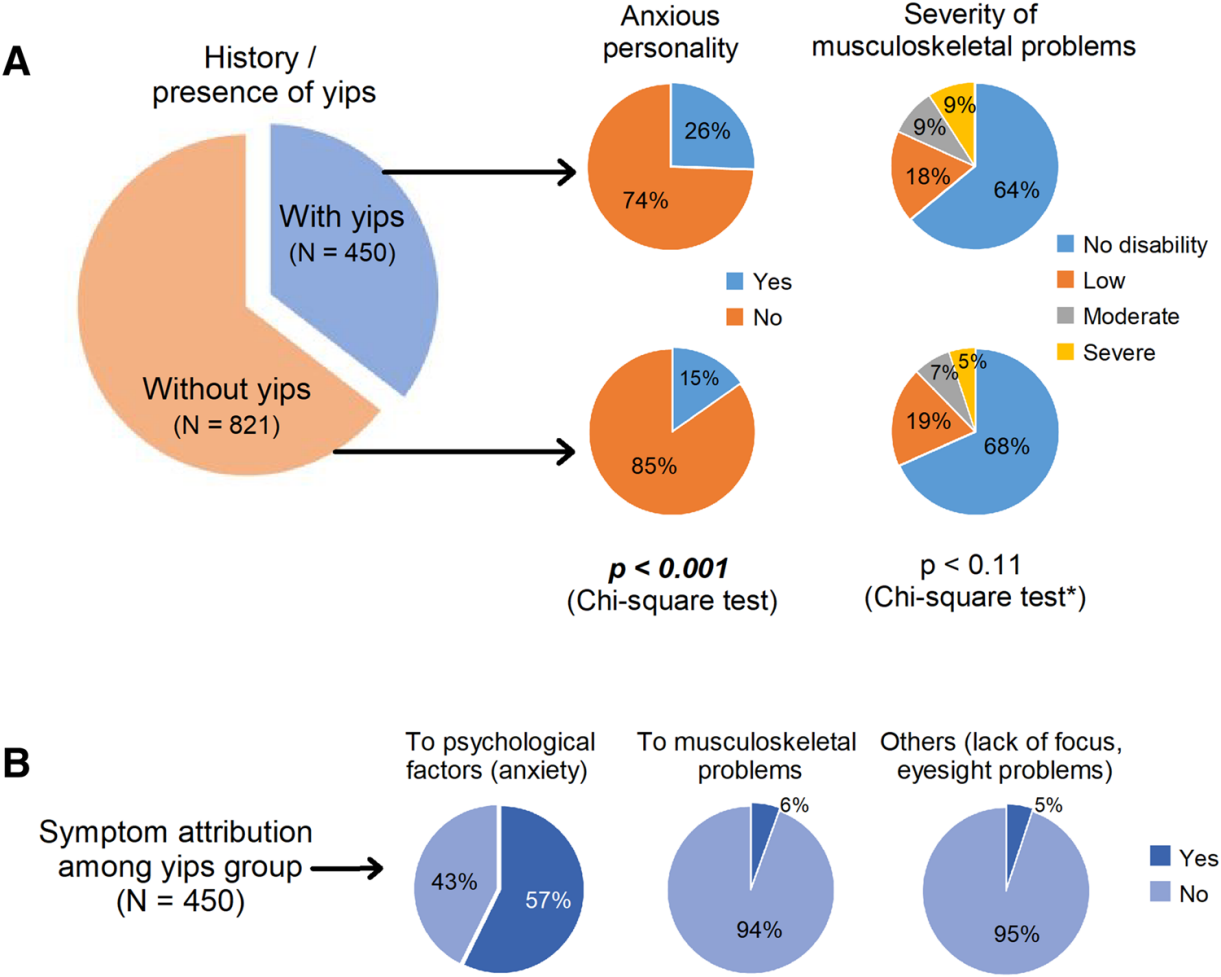
Response frequencies of golfers’ perception of yips. (**A**) Overview of classification of professional golfers. *Chi-square test represents the grading of at least one musculoskeletal problem (grades = none, low, moderate, severe) among the golfers. Significant differences were observed between normal and yips golfers having severe musculoskeletal problems (χ^2^ = 7.60, *p* = 0.006). (**B**) Degree of attribution of symptoms among respondents from yips-only group.

More golfers with yips answered that their personality was of nervous disposition (proportional test, chi-square test (χ^2^) = 20.25, *p* < 0.001). The responses for the possibility of at least a single musculoskeletal injury were similar between the groups (χ^2^ = 2.46, *p* = 0.117). However, yips golfers had a higher proportion of severe musculoskeletal problems (χ^2^ = 7.60, *p* = 0.006). When asked what the golfers felt their yips was due to, nearly 57% of yips golfers attributed the performance deficits to psychological causes. Only a meagre 5% felt yips was because of a movement-disorder, Fig. 2.

For subgroup analysis among yips-only respondents (N = 450), 29 golfers were excluded for one or more of the following reasons: (i) stopped playing golf altogether, (ii) did not specify any current symptomatology because of yips, or (iii) erroneous entries or unanswered questions; bringing the total number of yips-only respondents to N = 421.

**Figure 3:**
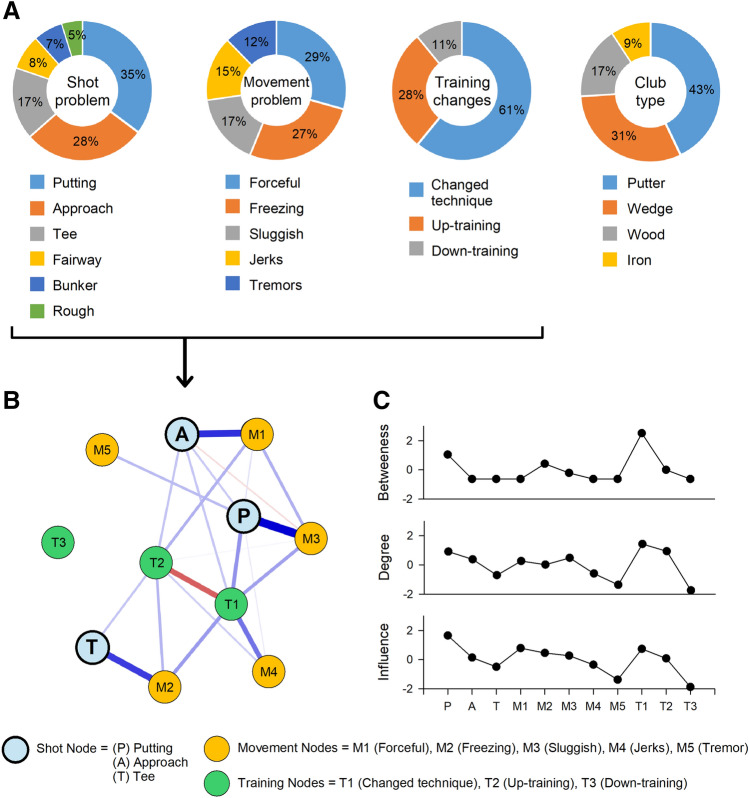
Frequency estimates, network and centrality plot for key factors associated with yips symptoms. (**A**) Frequency estimates of problem types and interventions in yips only golfers (N = 421). (**B**) Network structure for three variables (nodes) = shot-problem, movement-problem and training changes. Network edges were based on an Ising model (hyperparameter = 0.25) wherein blue edges represent positive relationship between the variables and red edges represent negative relationships. The strength of the connection between the nodes was proportional to the thickness of the edges represented between them. Only non-zero edges are shown, with sparsity = 0.56. The distance between the nodes was arbitrary, specified by a value given by the Fruchterman–Reingold algorithm (here = 0.7). The edge-weight stability demonstrating the robustness of the network is summarized in supplementary data. As seen from the network, putting (P), approach (A) and tee shots (T) nodes had strong edge connection to specific movement deficits (slowing, forceful and freezing of swing, respectively) in the network. (**C**) Centrality plot with respect to the network structure was explained via betweenness, degree and the explained influence of the variables (nodes) in the network. The X and Y axes specify the nodes and the indices respectively. Putting shots along with their predominant training patterns i.e. changing technique and increasing the frequency of training had strong degree centrality with significant influence on yips-golfers’ characteristics within the network.

Frequency estimates of problems with the use of club, the type of shots, the underlying movement deficit associated with it and any accompanying interventions for relief of symptoms are summarized in Fig. 3A. These variables were then grouped for network analysis to study their relationships (Fig. 3B). For shot-type, we focused on putting, approach and tee shots since they comprised the dominant shot problems (approx. 80%). Subsequent network estimation revealed the following key features: (i) a strong positive regression between putting and slowing of shots; between approach and forceful shots; and between tee shots and freezing of movement, (ii) a negative relationship between changing technique following yips and increasing the frequency of training, (iii) high degree centrality and influence of putting shots suggesting linkage between several other problematic movement-types, and (iv) high degree centrality and influence for changing technique and up-training compared to down-training for most golfers.

**Figure 4:**
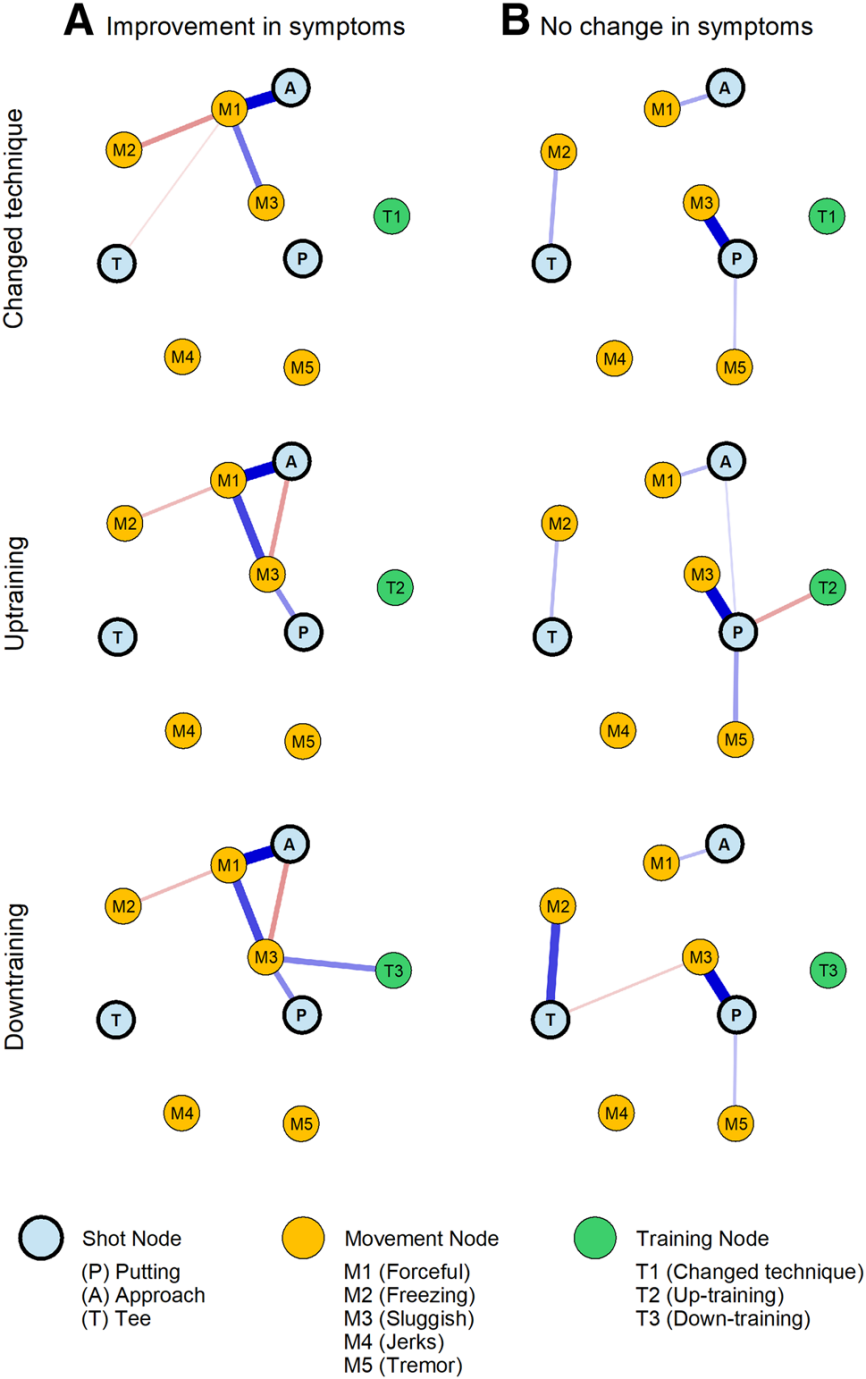
Network plots of outcome of interventions to relieve yips symptoms. In comparison to Fig. 3, the network plots were specified for each strategy that the golfers responded as: (**A**) An improvement in their symptoms or (**B**) No change in their symptoms. Due to the low sample sizes, the network construction was different from Fig. 3 in that (i) Golfers with worsening of symptoms were omitted, (ii) non-zero regression coefficients were specified with ‘OR’ rule, (iii) hyperparameter γ = 0, (iv) Fruchterman–Reingold distance = 1, and (v) network sparsity = 0.83. Network representations were constrained to a fixed ‘X’ and ‘Y’ axis coordinate system for visual comparison purposes. The resulting network showed that approach shots were more pliable for changes in self-administered training adjustments. On the other hand, golfers who reportedly did not show any change in symptoms were mostly with putting type of yips.

We then evaluated golfers’ strategies to counter the yips symptoms. Of the 421 respondents, only 253 answered if their techniques relieved (n = 102), worsened (n = 11) or showed no change (n = 140) in their symptoms. For network analysis, we excluded golfers whose symptoms worsened because of very low sample sizes. Network structures were extremely sparse, Fig. 4 (Sparsity index = 0.83). For each strategy type, golfers’ perception of improvement was seen only for approach shots. Those who reported no change in symptoms showed weaker connections between shot and the movement types, with putting shots being impervious to any improvements.

## Discussion

With a sizable response rate, our survey report provides a comprehensive overview of the perception of yips among Japanese golfers and quantifies the burden associated with it. In golfers who had the yips, we observed that (i) long golfing experience plays a crucial role in precipitating the symptoms of yips, (ii) kinematically, putting, approach and tee shots are frequently affected, in that order, each accompanied by a characteristic movement deficit, and (iii) whereas approach yips seems receptive to improvements via adjustments in their techniques or training patterns, the same does not hold true for putting or teeing yips in a majority of such golfers. Our findings also hint that despite reporting musculoskeletal involvement, a substantial number of yips-golfers perceive yips to be a psychological issue. This perception begets self-help strategies that appear futile in the management of yips without professional help.

Our results were congruent with several well-established findings observed in yips. Notably, the high proportion of yips golfers among older adults (50 to 64-year age group), a mean prevalence of yips among elite professionals at 35.4%, the probability of yips occurrence with long golfing exposure and the high predisposition to anxiety in such golfers, are in line with previously documented studies^2,3,18^. Notably, we observed that nearly 57% of yips-golfers attributed their symptoms to be psychological. We speculate that this perception among golfers may play a key role in creating a degree of anxiety that may impinge upon their gameplay. Models that explain sports related anxiety conceptualize that the cognitive self-evaluation and stress response if left unchecked, result in increased muscle tension, loss of focus and attention along with a range of other physiological behavioral changes^8^. This means that up to a certain point depending on the individual’s own threshold of sense of anxiety, the performance-anxiety loop can either streamline their quality of shots or might debilitate the task^37^. Anxiety tests should therefore serve as a baseline in any yips assessment with every golfer requiring the necessary appraisal to improve their performance.

On the other end of the yips spectrum are task-specific dystonia identified as involuntary excessive muscle contractions in repeatedly learnt skilled activity^10,23^. Given the overarching presence of performance anxiety among the participants in our survey, we observed that yips golfers had significantly higher musculoskeletal symptoms that affected not only their golfing performance, but also activities outside of their competitive environments. While traditionally, this does not fit into the definition of task-specific dystonia, one possible explanation for these findings is that these could be the same muscles that are required for executing day-to-day tasks. Given that symptoms of task-specific dystonia precipitate when there is continuous, intensive over-use of specific musculature for long periods of time^38^, these golfers are likely to be susceptible to movement disorders with or without performance anxiety^9^. This characteristic of dystonic yips golfers was also previous highlighted by Ioannou et.al. in which they argued that those with predominant motor instabilities often do not have strong psychological influence on their symptoms^11^.

Kinematic questionnaires revealed several distinct characteristics. Typically, professional golfers spend considerable practice time in perfecting the putting stroke^39^. We found that putting was the most affected stroke in yips-golfers, a finding that has been definitively reported in other studies as well^2,4,5^. The high degree centrality, noted via network analysis, showed that putting was accompanied by a variety of movement problems that resulted in abnormal control of swing i.e. forceful or slowing of the swing, or those suggestive of abnormal co-contractions i.e. tremors, jerks or freezing. Of the above, slowing of movement during putting was the most frequent. Approach and tee shots were characterized by forceful-strikes and freezing respectively. Considering the low degree and betweenness centrality for approach and tee shots, the above-mentioned movement problems for these types may be deemed specific.

Including training type within this network revealed certain important findings. First, both changing technique and up-training (increasing the frequency) had high degree centrality (positive edges to all movement-types) suggesting that golfers frequently tried one of these interventions. Second, the strong negative relationship between the two implied these strategies were mutually exclusive. Those who up-trained avoided changing their technique, a strategy seen frequently for approach or teeing yips. However, changing technique outweighed other modalities and considering its relationship with diverse muscular symptoms for putter’s yips, our findings reinforce the magnitude of performance deficits that golfers face in ameliorating putting symptoms.

Down-training showed no definitive relationship with other variables. Prior studies have demonstrated that down-training, along with retraining, have shown favorable outcomes regarding dystonic symptomatology^10,17,40–42^. Our findings may be a direct consequence of low sample sizes since the network construction in Ising models are critically dependent on it^32^. Still, it is vital to recognize why only about 10% of our respondents lowered their training habits. With high attribution rates (~ 57%) of yips to psychological causes, we speculate that this may simply be due to a (mis)conceptualization of the movement problem. Regardless, those who ascribe yips to a ‘choking’ phenomenon or perceive their crisis due to lack of practice are both at risk of overtraining and contributing to the abnormal plasticity^43^. It would therefore be necessary to sensitize the golfers about the benefits of rest and immobilization in rehabilitative retuning of the symptoms.

With the above self-administered strategies, network structures revealed that irrespective of the type of intervention, golfers’ perception of improvement was higher for approach shots than with putting or tee shots. Approach shots, in general, constitute the broadest category in golf in terms of distance from the putt^44^ and without causal evidence it may be difficult to conclude why such adaptability was seen among our golfers. Golfers with putter’s yips formed the biggest group who showed no changes in their symptoms. This may be a consequence of the diverse movement problems reported for putting shots. However, we wish to provide a conservative viewpoint for these outcomes since the networks were extremely sparse (along with bootstrapped confidence-interval overlaps), lowering the interpretations of the network structure.

In summary, it is crucial that golfers and trainers need to be mindful of yips phenomenon early in their commencement of training, especially for high-risk shots such as putting. This will enable golfers to be receptive to changes in technique so that new motor programs can be flexibly and successfully adopted if the yips do occur^5^. From our perspective, even when symptoms of yips are mild, in addition to the short-term fixes such as switching grips or changing clubs, it may be worthwhile for golfers to focus on intermittent training and alter training environments. The effects are expected to be twofold. First, any overload, strain or fatigue in the affected muscle groups would be countered using short ‘days-off’ from the training schedule. Second, this would favor relearning distance control and shot adjustments as a form of sensory retraining that is suggested to limit yips^4,5,13^. At this stage, golfers are recommended to have a close engagement with a professional coach and a medical practitioner to help monitor any worsening of symptoms that may affect grip strength, joint mechanics, or muscle co-contractions. This would allow golfers’ compliance towards prompt consults for electrophysiological lab diagnosis and medical management of the yips. These measures need not be restricted to golf, and could potentially be extended to other sports where yips is commonly encountered (e.g. baseball or cricket).

### ﻿Limitations

There are some limitations to our work. Only a few of the respondents (N = 6) from the survey had received a formal clinical diagnosis of task-specific dystonia. While the term ‘yips’ is relatively common among golfing circles, the knowledge of yips rested on the participant via seminars offered by the movement-disorder specialists. We were therefore careful in interpreting our findings without excessive speculation, since most respondents were not formally/physically diagnosed by a movement-disorder specialist. Given the high prevalence, we speculate a substantial number of golfers may have had mild-yips like behavior—inconsistencies during specific shots (e.g. Putting) or those that are not strictly confined to a specific type of shot^4^. To assertively characterize a stereotyped ‘movement disorder’ from a simple ‘movement disturbance’ it is essential to determine these features via visual/video examination by a movement-disorder specialist with or without neurophysiological testing in a task-based kinematic experimental paradigm. Furthermore, the yips spectrum comprises of several overlapping subtypes of movement disturbances^4,15,19^ that would require more detailed assessments in terms of cognitive status and longitudinal evaluations that were beyond the scope of our cross-sectional assessment.

As with any evaluation based on self-reports, our survey was likely to suffer from self-reporting bias^21^. With the information available over the internet, it is likely some respondents may have overestimated their problem. Contrarily, some symptoms may have been underreported because of a level of stigmata attached to the problem among athletes. Despite these subjective intricacies, we reckon that the golfers were well-informed about yips and therefore their responses were reasonable. We believe our method of assessment was in-line with prior studies with the sampling variations to be fair.

The survey detailed several movement-related problems in yips inadvertently creating a disproportionate focus wherein anxiety issues may not have been thoroughly assessed. Standardized anxiety tests such as sports competition anxiety test or state and trait anxiety tests, targeting golfers’ performance will be beneficial in revealing the role of anxiety and personality as moderator variables for yips. Addressing these aspects is likely to improve the quality of assessment in the future thereby allowing longitudinal comparisons as well as opening up to encompass athletes from other sports as well.

## Conclusions

Yips in golf has a stigma attached to it that is often problematic^24^. Whether the cause is anxiety or dystonia, the outcome is unitary—a distinct, appreciable loss in performance wherein mild cases may not be troublesome but as severity increases may essentially disrupt athletes’ careers. Fortunately, most professional Japanese golfers are unaffected by the yips. While our report highlighted the characteristics, features and similarities with several other prior studies on yips, there were some observations among Japanese golfers that necessitates deliberations. Despite the musculoskeletal and/or kinematic issues, many yips-golfers speculate their condition to be psychological. With no apparent benefits owing to training changes, it may be crucial to target golfers early in their career with an assertive informational outreach regarding the movement-disorder aspect of yips. For health-care providers, it will be imperative to address these challenges to influence golfers’ compliance towards accessing timely remedies for their symptoms.

## Supplementary Information

Supplementary Information.

## Data Availability

Data that support the findings of this study will be made available from the corresponding author upon reasonable request.
