# Supplementary material for: Perception of yips among professional Japanese golfers: perspectives from a network modelled approach
Source: Sci Rep. 2021 Oct 11;11:20128. doi: 10.1038/s41598-021-99128-9 (PMC8505642; doi:10.1038/s41598-021-99128-9)
Supplement: Supplementary file 1 — Supplementary Information. [file 41598_2021_99128_MOESM1_ESM.docx]

**Supplementary Data**

**Perception of yips among professional Japanese golfers: perspectives from a network modelled approach**

**Gajanan S. Revankar^1,4^, Yuta Kajiyama^1^, Yasufumi Gon^1^, Issei Ogasawara^2^, Noriaki Hattori^1,5^, Tomohito Nakano^1^, Sadahito Kawamura^3^, Yoshikazu Ugawa^6^, Ken Nakata^2*^ and Hideki Mochizuki^1*^**

^1^Department of Neurology, Graduate School of Medicine, Osaka University, Osaka, Japan

^2^Department of Health and Sport Sciences, Graduate School of Medicine, Osaka University, Osaka, Japan

^3^Department of Orthopedics, Graduate School of Medicine, Osaka University, Osaka, Japan

^4^Institute for Transdisciplinary Graduate Degree Programs, Osaka University, Osaka, Japan

^5^Department of Rehabilitation, Faculty of Medicine, Academic Assembly, University of Toyama, Toyama, Japan

^6^Department of Human Neurophysiology, Fukushima Medical University, Fukushima, Japan

**Supplementary Figure-1: Edge weight stability**

**
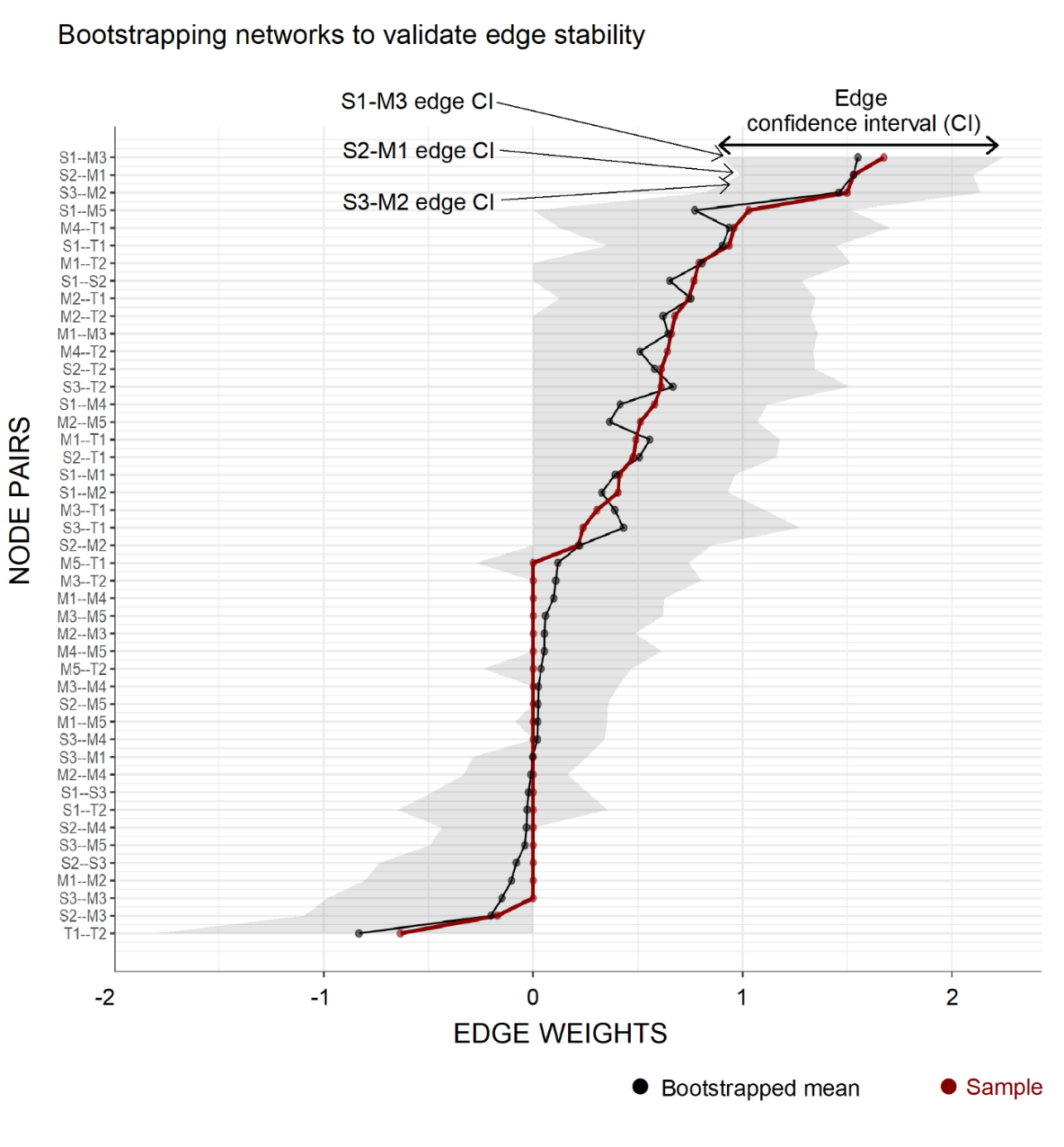
**

Supplementary Figure-1 caption: Graph shows between-nodes edge weight confidence interval (CI) for sample and bootstrapped means. Red scatter-line plots represent original edge weight of the network. Black scatter-line plot denotes bootstrapped edge weights of random networks. The random networks were created by bootstrapping 1000 times. Edge CI which did not overlap with other nodes of interest were considered significant. Notably, edge CI for putting (S1), approach (S2) and tee shots (S3) pairs with their corresponding musculoskeletal problems (M3, M1 and M2 respectively) were significantly different with other shot-movement problem pairs.
